# Grazing intensity changed the activities of nitrogen assimilation related enzymes in desert *Steppe Plants*

**DOI:** 10.1186/s12870-021-03205-0

**Published:** 2021-09-25

**Authors:** Aimin Zhu, Haili Liu, Yuehua Wang, Hailian Sun, Guodong Han

**Affiliations:** 1 College of Grassland, Resources and Environment, Key Laboratory of Grassland Resources of the Ministry of Education of Chian, Key Laboratory of Forage Cultivation, Processing and Higher Efficient Utilization of the Ministry of Agriculture and Rural Affaires of China, Inner Mongolia Key Laboratory of Grassland Management and Utilization, Inner Mongolia Agricultural University, 010019 Hohhot, China; 2 Research Base of Academy of Agriculture and Animal Husbandry of Inner Mongoloia, 010031 Hohhot, China

**Keywords:** Grazing, *Stipa breviflora*, Nitrogen assimilation, Enzyme activity

## Abstract

**Background:**

Nitrogen, as a limiting factor for net primary productivity in grassland ecosystems, is an important link in material cycles in grassland ecosystems. However, the nitrogen assimilation efficiency and mechanisms of grassland plants under grazing disturbance are still unclear. This study investigated *Stipa breviflora* desert steppe which had been grazed for 17 years and sampled the root system and leaf of the constructive species *Stipa breviflora* during the peak growing season under no grazing, light grazing, moderate grazing and heavy grazing treatments. The activities of enzymes related to nitrogen assimilation in roots and leaves were measured.

**Results:**

Compared with no grazing, light grazing and moderate grazing significantly increased the activities of nitrate reductase (NR), glutamine synthetase (GS), glutamic oxaloacetic transaminase (GOT) and glutamic pyruvate transaminase (GPT) in leaves, and GS, GOT and GPT in roots of *Stipa breviflora*, while heavy grazing significantly decreased the activities of GS in leaves and NR in roots of *Stipa breviflora*. NR, GOT and GPT activities in leaves and roots of *Stipa breviflora* were positively correlated with nitrogen content, soluble protein, free amino acid and nitrate content.

**Conclusions:**

Grazing disturbance changed the activities of nitrogen assimilation related enzymes of grassland plants, and emphasized that light grazing and moderate grazing were beneficial for nitrogen assimilation by grassland plants. Therefore, establishing appropriate stocking rates is of great significance for material flows in this grassland ecosystem and for the stability and sustainable utilization of grassland resources.

## Introduction

Grazing is considered to be an important measure in grassland management. However, with the intensification of human activities, grazing disturbance in grasslands has become a concern [1, 2], and livestock grazing management remains very important for future grassland protection [3]. Previous studies have shown that grazing affects grassland ecosystems mainly by changing plant and soil properties [4–6]. Desert grassland accounts for 39 % of the grassland in Inner Mongolia [7], and long-term overgrazing has exacerbated grassland degradation in recent years, causing decreases in vegetation coverage, biodiversity and productivity [8]. Therefore, it is very important to understand how grazing management affects grassland ecosystems, especially in desert grasslands. Kotanen et al. [9] have shown that grazing can lead to decreases in nutrient content and leaf area of plants, thus affecting plant photosynthesis. Many previous studies have reported effects of grazing on nitrogen uptake and utilization of plants [10–12], but these studies have mainly focused on the level of nitrogen stoichiometry, and relatively few studies have focused on the mechanisms through which grazing affects nitrogen assimilation by plants.

**Fig. 1:**
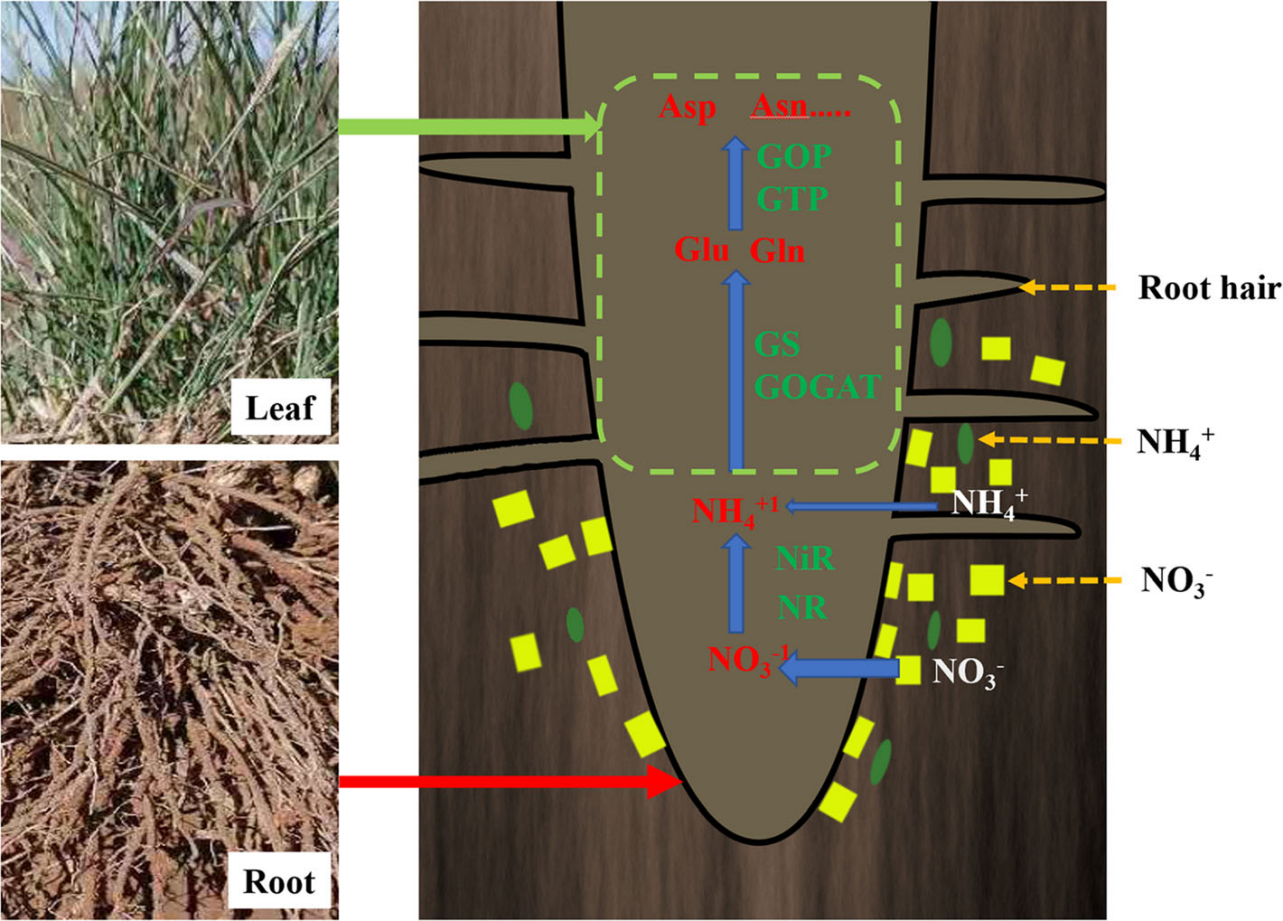
Schematic diagram of plant nitrogen absorption and nitrogen assimilation process. The picture on the left shows the roots and leaves of plants, and the picture on the right shows the process of plant roots absorbing No_3_^−^ and NH_4_^+^ in soil and nitrogen assimilation catalyzed by enzymes. NR: nitrate reductase; NiR: nitrite reductase; GS: glutamine synthetase; GOGAT: glutamic acid synthetase; GOT: Glutamic oxaloacetic transaminase; GPT: glutamic pyruvate transaminase; Glu: glutamate; Gln: glutamine; Asp: Aspartic; Asn: Asparagines

Nitrogen is one of the essential elements for plant growth. Nitrogen assimilation refers to the process by which plants absorb nitrogen from the environment and synthesize nitrogen-containing organic compounds such as amino acids and proteins, a process in which many enzymes participate (Fig. 1). Inorganic nitrogen sources (ammonium salt and nitrate) in soil are the main nitrogen sources of plants. Although most plants can absorb NH_4_^+^, NO_3_^−^ is the main nitrogen source for plants. The plants mainly rely on the cortex cells of the root to absorb nitrate from the rhizosphere soil. The leaves of plants can also absorb nitrate, but the amount of nitrate absorption is smaller than root absorption. In the process of nitrate assimilation, NO_3_^−^ is reduced to NH_4_^+^ by nitrate reductase and nitrite reductase [13], and then assimilated to glutamic acid and glutamine by glutamic acid synthase [14, 15]. After that, other amino acids or nitrogenous compounds are formed under the action of transaminase. Glutamine synthetase (GS) and glutamic acid synthetase (GOGAT) are two important enzymes involved in catalysis during the glutamic acid synthetase cycle. The two important enzymes involved in the catalysis in the process of ammonia transfer are glutamic pyruvate transaminase (GPT) and glutamic oxaloacetic transaminase (GOT) [16, 17]. Research on the mechanisms of plant nitrogen assimilation is relatively mature, and has mainly been conducted on wheat, rice, corn, soybean and other major crops [18–20]. However, there are few studies on the mechanism of plant nitrogen assimilation in grazing grassland.

The soil of *Stipa breviflora* desert steppe is poor and the natural conditions are harsh. Species competition caused by grazing is also reflected in competition for nutrient resources [7, 21]. In order to adapt to the environment, species gradually form external morphological characteristics and internal physiological adaptation strategies [22, 23]. The plant material selected in this study was *Stipa breviflora*, which is a constructive species in desert steppe. The population characteristics and morphological characteristics of *S. breviflora* change greatly under long-term grazing at different grazing intensities. For example, with increasing grazing intensity, individuals of *S. breviflora* undergo dwarfing, as leaves and plant clusters become shorter and aboveground biomass decreases, although the importance value increases with increasing grazing intensity and relative coverage and density also increase significantly [24]. Smith [25] and Li et al. [26] have come to similar conclusions in other grazing grassland studies. Previous studies showed that the total nitrogen content in plant roots and leaves changed significantly under different grazing treatments [7, 27, 28].

However, it is not clear whether the changes in total nitrogen content in roots and leaves of *S. breviflora* under different grazing intensities are related to changes in the enzyme activities involved in nitrogen assimilation. Therefore, we proposed two scientific hypotheses: (1) Different grazing intensities change the activities of nitrogen assimilation related enzymes in leaves and roots of *S. breviflora*, which is consistent with the moderate disturbance hypothesis (i.e., the activities of nitrogen assimilation related enzymes in leaves and roots of *S. breviflora* would be higher under moderate grazing intensities). (2) The contents of nitrogen and nitrogen compounds in leaves and roots of *S. breviflora* are closely related to the activities of enzymes related to nitrogen assimilation, and show a significant positive correlation. In order to test these hypotheses, we sampled the constructive species *S. breviflora* in desert steppe experimental plots with 17 years grazing history in the peak growing season of 2020, and measured and analyzed the related indicators. This study can contribute to understanding of the patterns and mechanisms of nutrient absorption and utilization of grassland plants, and also provide technical and theoretical guidance for the rational and sustainable utilization of grazed grassland.

## Methods

### Plant materials and sources

*S. breviflora* samples were collected in long-term grazing experimental plots, which is located in Siziwang Banner (41 ° 47’17 “N, 111 ° 53’46” E, elevation 1 450 m) at the comprehensive experiment and demonstration center of Inner Mongolia Academy of Agriculture and Animal Husbandry Sciences, China. *S. breviflora* is a wild constructive species in desert grassland that is widely distributed in the western part of Inner Mongolia Autonomous Region, and it is not an endangered plant species. The manager of Inner Mongolia Academy of Agriculture and Animal Husbandry Sciences approved us to collect samples of *S. breviflora*, and identified the samples and supervised the sampling process. The annual average precipitation of desert steppe is 280 mm, more than 80 % of which is concentrated in May to September, and the average annual temperature is 3.4 ℃. The soil type is light chestnut soil.

### Experimental design

The experiment was a randomized block experiment. The experimental plots covered about 50 hm^2^ of natural grassland, and had been enclosed for 17 years (2004–2020). The plots were divided into three blocks. Each block had four treatment plots (no grazing, light grazing, moderate grazing and heavy grazing). Each treatment was repeated three times, and the area of each experimental plot was 4.4 hm^2^. The stocking rate in each treatment was 0 (no grazing, CK), 0.91 (light grazing, LG), 1.82 (moderate grazing, MG) and 2.71 (heavy grazing, HG) sheep unit · (hm^2^ A^− 1^) ^− 1^. The stocking rates were set according to the results of Wei et al. [29]. In this experiment, 2-year-old adult sheep were selected and allowed to graze from June to November every year. During the experiment, the management measures in each grazing area were the same. The sheep were driven into the grazing area at 6 a.m. and back to the shed at 6 p.m. every day, during which the sheep were free to feed. Water was provided twice a day in the morning and evening, and supplementary salt was regularly available in the form of salt bricks.

### Sampling and measurements

Sampling of the roots and leaves of *Stipa breviflora*: sampling was conducted on August 10, 2020. A transect was set passing through the center of the study plot, and six sampling points with a spacing of 50 m were arranged on the transect. Five clumps of plants were randomly selected around each sampling point to dig the plant roots 20 cm deep underground and loose soil on the surface of the roots was removed, and the rhizosphere soil was obtained by the shaking method. After the aboveground green leaves and roots had been separated, they were immediately put into an ice box, and samples from every three sampling points were combined into one sample. The roots and leaves from each of the 12 treatment plots were sampled with 6 replicates, making a total of 48 samples. Half of the samples were used to determine the activities of nitrogen assimilation related enzymes and nitrogen compounds in roots and leaves. Half of the samples were heated at 105 ℃ for 30 min and then dried at 65 ℃ for 24 h to determine the total nitrogen content. Five replicates of each treatment sample were selected for determination of nitrogen assimilation related enzyme activities and nitrogen compounds. The remaining samples and the remaining set of replicates were stored at − 80 ℃ in a refrigerator as a backup. The contents of total nitrogen in rhizosphere soil, plant roots and leaves were determined using an elemental analyzer.

We cleaned the fresh plant leaves and roots repeatedly with distilled water until there is no dirt, and then absorbed the residual distilled water on the surface of the leaves and roots with filter paper. The relevant indicators were determined according to the experimental guidance as follows.

#### Nitrate nitrogen

We put 2.00 g of chopped and mixed plant samples into a test tube, added 10 ml of non-ionic water, sealed the tube, and placed it in a boiling water bath for 30 min, filtered the extract into a 25 ml volumetric flask, and fixed the volume to the scale. In a 1 ml sample solution, we added 0.4 ml 5 % salicylic acid sulfuric acid solution, mixed it well, and left it at room temperature for 20 min, added 9.5 ml 8 % NaOH solution, and then measured and record the optical density at 410 nm wavelength, and calculated the content of nitrate nitrogen [30].

#### Soluble protein

0.5 g of plant sample was ground and extracted with 5 ml distilled water. We took 1.0 ml and put it into a tube with a plug, added 5 ml of Coomassie brilliant blue G-250 solution, mixed it well, and after 2 min measured and recorded the optical density value at 595 nm wavelength, and calculated the soluble protein content [30].

#### Proline

We put 0.5 g of chopped and mixed plant sample into a test tube, added 5 ml of 3 % sulfosalicylic acid solution, sealed the test tube, and extracted it in boiling water bath for 10 min. 2 ml of the extraction solution was put into a test tube, and 2 ml glacial acetic acid and 3 ml chromogenic solution (glacial acetic acid and 6 mol L^− 1^ phosphoric acid mixed in 3:2) were added before heating in boiling water bath for 40 min. Then, we added 5 ml toluene for extraction, determined and recorded the absorbance at 520 nm, and calculated the content of proline [30].

#### Free amino acids

We took 1.00 g of plant sample, put it in a mortar, added 5 ml of 10 % acetic acid, ground it thoroughly, transferred it to a 100 ml volumetric flask, fix the volume with distilled water, and filtered it. We put 0.5 ml of sample solution into a 20 ml plug calibration tube, added 0.5 ml of acetic acid cyanate buffer solution and 0.5 ml of 3 % ninhydrin solution, put it in boiling water bath for 12 min, added 5 ml of 95 % ethanol after cooling, and then sealed and shook the tube vigorously, after which we measured and recorded the optical density at 560 nm, and calculated the concentration of free amino acids [30].

#### Nitrate reductase (NR) activity

0.5 g fresh plant samples were cut up in a mortar and frozen for 30 min at low temperature. 4 ml buffer solution (0.1211 g cysteine, 0.0372 g EDTA dissolved in 100 ml 0.025 mol · L^− 1^ pH 8.7 phosphate buffer solution) was added to the mortar, and then transferred to a centrifuge tube and centrifuged at 4 ℃ 4000r min^− 1^ for 15 min. We put 0.4 ml crude enzyme solution in a 10 ml test tube, added 1.4 ml 0.1 mol L^− 1^ KNO_3_ phosphate buffer and 0.2 ml NADH solution, mixed it well, and kept it warm in a water bath at 25 ℃ for 30 min, and a control without NADH was replaced with 0.1 mol L^− 1^ pH 7.5 phosphate buffer. After heat preservation, we added 1 ml of sulfonamide solution and 1 ml of naphthyl vinylamine solution immediately. After 15 min of color development and centrifuging at 4000r min^− 1^ for 5 min, we took the supernatant, measured and recorded the optical density at 540 nm wavelength, and calculated the nitrate reductase activity [30].

#### Glutamine synthetase (GS) and glutamic acid synthetase (GOGAT)

We put 1.0 g of plant material into a mortar, added 6 ml buffer (0.05 mol L^− 1^ Tris-HCl buffer, pH 8.0), put it in an ice bath, ground it evenly, transferred it into a centrifuge tube, centrifuged at 4 ℃ 15000r min^− 1^ for 20 min, and the resulting supernatant was crude enzyme. For **GS**, we combined 1.6 ml of reaction solution B (0.1 mol L^− 1^ Tris-HCl buffer, 80 mol L^− 1^ hydroxylamine hydrochloride, pH 7.4), 0.7 mL of crude enzyme solution and 0.7 mL of 50 mmol L^− 1^ ATP solution, mixed it well, kept it at 37 ℃ for 0.5 h, added 1 ml of chromogenic agent, centrifuged at 5000r min^− 1^ for 10 min, and took the supernatant to determine the absorbance at 540 nm and recorded it, and using 1.6 ml of mixture A (0.1 mol L^− 1^ Tris-HCl buffer, pH 7.4) as the control, the activity of glutamine synthetase was calculated according to the difference in absorbance between reaction solution B and reaction solution A. For **GOGAT**, the total volume of the reaction was 3 ml (0.4 ml 20 mmol L^− 1^ L-glutamine, 0.05 ml 0.1 M ɑ-ketoglutarate, 0.1 ml 10mmol l-1kcl, 0.2 ml 3 mmol L^− 1^ NADH, and 0.5 ml enzyme solution), and the insufficient volume was supplemented by 25 mmol l L^− 1^ pH 7.6 Tris HCl buffer. The reaction was started by L-glutamine, and the change in nitrification value at 340nm wavelength was determined. A decrease of 0.001 extinction value per minute was taken as an enzyme activity unit [31, 32].

#### Glutamic oxaloacetic transaminase (GOT) and glutamic pyruvate transaminase (GPT)

We put 0.2 g of the mixed plant material into a mortar, added 2.0 ml buffer solution (0.05 mol L^− 1^ Tris-HCl buffer solution, pH 7.2), ground it in an ice bath, and the homogenate was centrifuged at 20,000×g for 20 min. The supernatant was crude enzyme solution. For **GOT**, we took two 10 ml test tubes. One tube was used as the test tube, and we added 0.1 ml of crude enzyme and 0.5 ml of GOT substrate solution. The other tube was used as the control tube, and we added 0.1 ml of crude enzyme. The two test tubes were placed in 37 ℃ water bath for 60 min at the same time, and then removed. We added 0.5 ml of 2,4-dinitrophenylhydrazine solution to each test tube to terminate the reaction. We added 0.5 ml of GOT substrate solution to the control test tube and put the two test tubes in 37 ℃ water bath for 20 min, took them out, and then added 0.4 mol L^− 1^ NaOH 5.0 ml to each tube, mixed them well, then compared the color with 500 nm wavelength after 10 min, adjusted the zero point with distilled water, and read the absorbance value and calculated the GOT activity. **For GPT**, we took two 10 ml test tubes. One tube was used as the test tube, and we added 0.1 ml of crude enzyme and 0.5 ml of GPT substrate solution. The other tube was used as the control tube, and we added 0.1 ml of crude enzyme. The two test tubes were placed in 37 ℃ water bath for 30 min at the same time, and then removed. We added 0.5 ml of 2,4-dinitrophenylhydrazine solution to each test tube to terminate the reaction. We added 0.5 ml of GPT substrate solution to the control test tube, and put the two test tubes in 37 ℃ water bath for 20 min, removed them, and then added 0.4 mol L^− 1^ NaOH 5.0 ml to each tube and mixed them well. We compared the color with 500 nm wavelength after 10 min, adjusted the zero point with distilled water, and read the absorbance value and calculated the GPT activity [33].

#### Ammonium nitrogen and nitrate nitrogen in rhizosphere soil

Ammonium nitrogen and nitrate nitrogen content in rhizosphere soil were extracted by potassium chloride and determined by AA3 continuous flow analyzer (AVTO ANACY AA3, German SEAL company).

#### Nitrogen content in roots and leaves

The dried plant samples were ground into powder by ball mill, and the nitrogen content in leaves and roots was determined by elemental analyzer (Elementar Vario MACRO CUBE).

### Statistical analyses

**Fig. 2:**
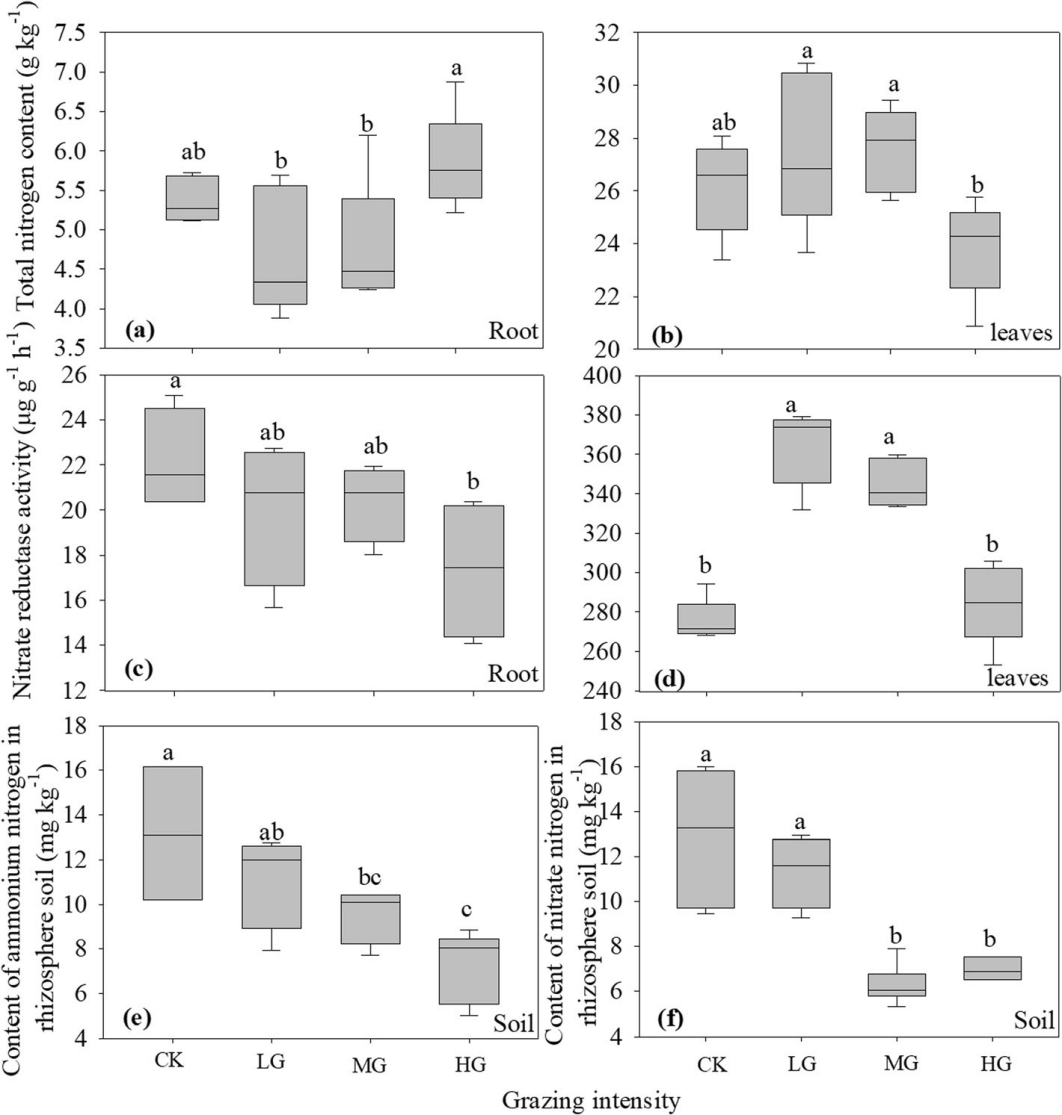
Effects of grazing intensity on total nitrogen content, nitrate reductase activity in roots and leaves of *Stipa breviflora* and contents of ammonium nitrogen and nitrate nitrogen content in rhizosphere soil of *Stipa breviflora*. **a** and **b** total nitrogen content (g kg^− 1^), **c** and **d** nitrate reductase activity (µg g^− 1^ h^− 1^), **e** ammonium nitrogen content (mg g^− 1^), **f** nitrate nitrogen content (mg kg^− 1^). Different lowercase letters indicate significant difference at *P*<0.05. CK, LG, MG and HG refer to no grazing, light grazing, moderate grazing and heavy grazing, respectively

**Fig. 3:**
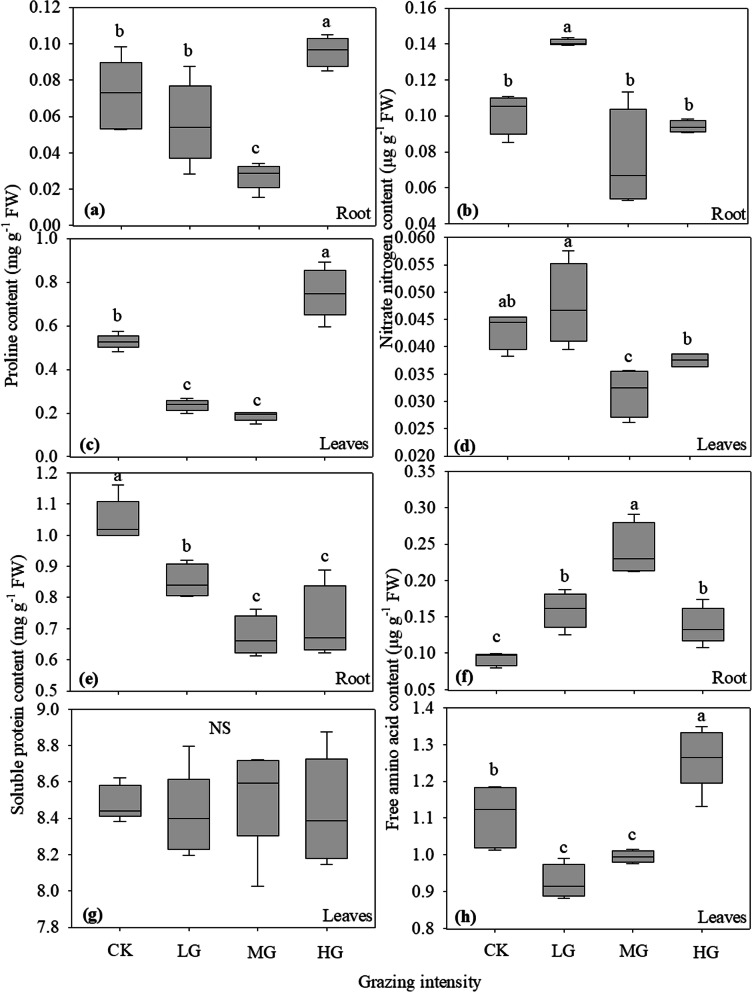
Effects of grazing intensity on nitrogen compounds and nitrate nitrogen contents in roots and leaves of *Stipa breviflora*. **a** and **c** proline content (mg g^− 1^ FW), **b** and **d** nitrate nitrogen contents (µg g^− 1^ FW), **e** and **g** soluble protein content (mg g^− 1^ FW), **f** and **h** free amino acid content (µg g^− 1^ FW). Different lowercase letters indicate significant difference at *P*<0.05. “Ns” in the figure indicates that there is no significant difference among grazing treatments. CK, LG, MG and HG refer to no grazing, light grazing, moderate grazing and heavy grazing, respectively

**Fig. 4:**
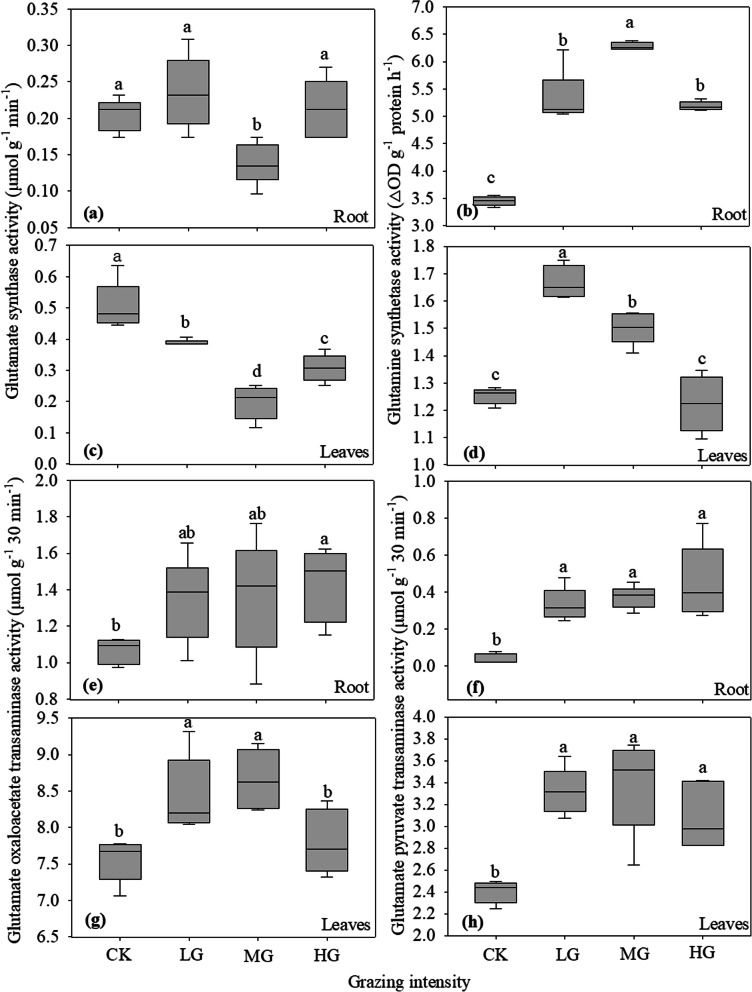
Effects of grazing intensity on activities of nitrogen assimilation related enzymes in roots and leaves of *Stipa breviflora*. **a** and **c** glutamate synthase activity (µmol g^− 1^ min^− 1^), **b** and **d** glutamine synthetase activity (△OD g^− 1^ protein h^− 1^), **e** and **g** glutamic oxaloacetic transaminase activity (µmol g^− 1^ 30 min^− 1^), **f** and **h** glutamate pyruvate transaminase activity (µmol g^− 1^ 30 min^− 1^). Different lowercase letters indicate significant difference at *P*<0.05. CK, LG, MG and HG refer to no grazing, light grazing, moderate grazing and heavy grazing, respectively

**Fig. 5:**
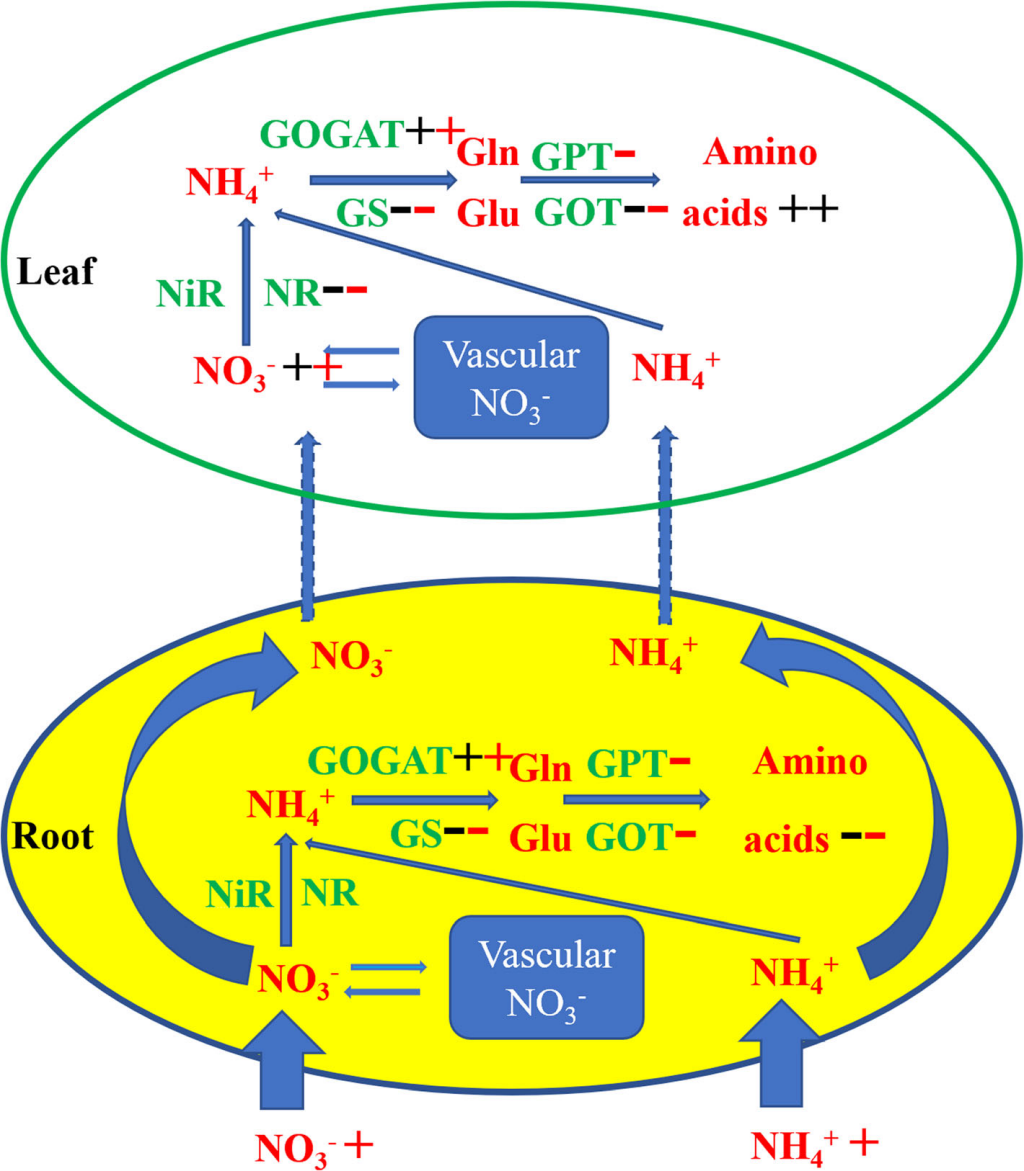
Effects of heavy grazing and no grazing on nitrogen absorption and assimilation pathways in roots and leaves of *S. breviflora*. The black “+” and “-“ in the figure represent the significant positive and negative effects of heavy grazing on the index compared with moderate grazing, and the red “+” and “-“ represent the significant positive and negative effects of no grazing on the index compared with moderate grazing. NR: nitrate reductase; NiR: nitrite reductase; GS: glutamine synthetase; GOGAT: glutamic acid synthetase; GOT: Glutamic oxaloacetic transaminase; GPT: glutamic pyruvate transaminase; Glu: glutamate; Gln: glutamine

One-way ANOVAs with Duncan’s test were used to evaluate the difference between the four grazing intensities, and P < 0.05 was used to indicate significant difference. We used SPSS25.0 software to calculate Pearson correlation coefficients between variables (correlations between nitrogen content, nitrogen compound content and activities of enzymes related to nitrogen assimilation), * and ** are used to denote significant and extremely significant correlations, respectively (Table 1). Figures 2, 3 and 4 were plotted with Sigma Plot 14.0 software. Taking moderate grazing as the standard, we compared the activities of enzymes related to nitrogen assimilation in plants from no grazing and heavy grazing treatments, and plotted the changes in enzyme activities in the process of nitrogen assimilation with PowerPoint software (Fig. 5).

## Results

The total nitrogen content of the root system of *S. breviflora* was significantly higher than that under light grazing and moderate grazing, while in the heavy grazing treatment the total nitrogen content in leaves was significantly lower than that under light grazing and moderate grazing (Fig. 2a and **b**). The results showed that heavy grazing increased the total nitrogen content in the roots and decreased the total nitrogen content in the leaves of *S. breviflora* to a certain extent, especially under heavy grazing. Nitrate reductase plays an important role in the conversion of NO_3_^−^ to NH_4_^+^ in plants. The activity of nitrate reductase in roots under heavy grazing was the lowest, which was significantly lower than that under no grazing, while there was no significant difference between light and moderate grazing (Fig. 2c**)**. Compared with light and moderate grazing, no grazing and heavy grazing significantly reduced nitrate reductase activity in leaves of *S. breviflora*. (Fig. 2d). It shows that light grazing and moderate grazing are conducive to the transformation of NO_3_^−^ into NH_4_^+^ in plant roots and leaves.

The nitrogen absorbed and assimilated by plants mainly came from ammonium and nitrate in rhizosphere soil. Our study showed that grazing intensity had a significant effect on the content of ammonium and nitrate in the roots of *S. breviflora*. With increasing grazing intensity, the content of ammonium nitrogen in the rhizosphere soil of *S. breviflora* decreased gradually (Fig. 2e). The nitrate content in the rhizosphere soil of *S. breviflora* under moderate and heavy grazing treatments was significantly lower than that under no grazing and light grazing treatments (Fig. 2f).

Nitrogen compounds in plants include soluble proteins and various amino acids. This study shows that grazing has affected the content of nitrogen compounds in plant roots and leaves (Fig. 3). Grazing intensity had a similar effect on the proline content in roots and leaves of *S. breviflora*, which decreased first and then increased with increasing grazing intensity (Fig. 3a and **e**). The proline content was the lowest under moderate grazing, and the highest in the heavy grazing treatment, and the proline content in the heavy grazing treatment was significantly higher than that in no grazing, light and moderate grazing treatments. The content of nitrate nitrogen in the roots and leaves of *S. breviflora* under moderate and heavy grazing was significantly lower than that under light grazing (Fig. 3b and **d**). The content of soluble protein in the roots of *S. breviflora* under grazing prohibition was significantly higher than that under light, medium and heavy grazing, and the content of soluble protein under light grazing was significantly higher than that under medium and heavy grazing (Fig. 3e). There was no significant difference in soluble protein content of *S. breviflora* leaves under different grazing treatments (Fig. 3 g). With increasing grazing intensity, the content of free amino acids in the roots of *S. breviflora* increased at first and then decreased, and was the highest under moderate grazing and the lowest in the no grazing treatment (Fig. 3f). However, free amino acids in leaves first decreased and then increased, and was the lowest under light grazing and the highest under heavy grazing (Fig. 3 h).

GS / GOGAT cycle is an important process in plant nitrogen assimilation, in which glutamate synthase and glutamine synthetase catalyze the conversion of NH_4_^+^ to glutamate and glutamine. The activity of glutamate synthase in roots and leaves of *S. breviflora* under moderate grazing was significantly lower than that under no grazing, light grazing and heavy grazing, and the activity of glutamate synthase in leaves under light and heavy grazing was significantly lower than that under no grazing (Fig. 4a and **c**). With increasing grazing intensity, the activity of glutamine synthetase in roots and leaves of *S. breviflora* first increased and then decreased (Fig. 4b and **d**).

Glutamic oxaloacetic transaminase and glutamate pyruvate transaminase play an important role in catalyzing the conversion of glutamate and glutamine to aspartic and asparagines. The effects of grazing intensity on the activity of glutamic oxaloacetic transaminase in the roots and leaves of *S. breviflora* differed. The activity of glutamic oxaloacetic transaminase in the roots of *S. breviflora* under heavy grazing was significantly higher than that under no grazing, while the activity of glutamic oxaloacetic transaminase in the leaves of *S. breviflora* under light and moderate grazing was significantly higher than that under no grazing and heavy grazing (Fig. 4e and **g**). Grazing had the same effect on the activity of glutamate pyruvate transaminase in the roots and leaves of *S. breviflora*. There was no significant difference among light, moderate and heavy grazing, but they were significantly higher than in the no grazing treatment (Fig. 4f and **h**).

According to Table 1, the activities of glutamine synthetase, glutamic oxaloacetic transaminase and glutamic pyruvate transaminase were significantly negatively correlated with the total nitrogen content in roots and leaves, while the activities of nitrate reductase, glutamic oxaloacetic transaminase and glutamic pyruvate transaminase were significantly positively correlated with total nitrogen content. The content of soluble protein was positively correlated with the activities of glutamine synthetase, nitrate reductase, glutamic oxaloacetate transaminase and pyruvate transaminase. Nitrate nitrogen content in roots and leaves was significantly positively correlated with glutamate synthetase activity, while nitrate nitrogen and free amino acid content were significantly negatively correlated with glutamine synthetase activity. The contents of nitrate nitrogen and free amino acids were positively correlated with the activities of nitrate reductase, glutamic oxaloacetic transaminase and glutamic pyruvate transaminase.

**Table 1:** Correlation analysis of nitrogen content, nitrogen compound content and nitrogen assimilation related enzyme activities

Indexes	TN	SP	Pro	NN	FAA
GOGAT	0.665	0.674	0.656	0.789*	0.652
GS	-0.922**	-0.937**	-0.744*	-0.816*	-0.891**
GOT	0.996**	0.994**	0.688	0.871**	0.961**
GPT	0.978**	0.975**	0.66	0.856**	0.945**
NR	0.992**	0.984**	0.629	0.893**	0.933**

Note: GOGAT, GS, GOT, GPT and NR represent glutamate synthetase, glutamine synthetase, glutamate oxaloacetate transaminase, glutamate pyruvate transaminase and nitrate reductase, respectively. TN, SP, Pro, NN and FAA represent total nitrogen content, soluble protein, proline, nitrate nitrogen and free amino acid, respectively. “* *” indicates significant correlation at 0.01 level and “*” indicates significant correlation at 0.05 level. “-“indicates significant negative correlation at 0.01 or 0.05 level

Taking moderate grazing as the standard, we compared the activities of enzymes related to nitrogen assimilation in plants from no grazing and heavy grazing treatments. The activities of enzymes related to nitrogen assimilation in roots and leaves of *S. breviflora* changed to different degrees under grazing exclusion and heavy grazing (Fig. 5). Although the content of nitrate and ammonium nitrogen in the rhizosphere soil of *S. breviflora* was high under grazing exclusion, the activities of nitrate reductase, glutamine synthetase, glutamic oxaloacetic transaminase and glutamic pyruvate transaminase in leaves of *S. breviflora* under grazing exclusion were low, and the activities of glutamine synthetase, glutamic oxaloacetic transaminase and glutamic pyruvate transaminase in roots of *S. breviflora* under grazing exclusion were low. Heavy grazing decreased the activities of nitrate reductase, glutamine synthetase and glutamic pyruvate transaminase in leaves of *S. breviflora*, and the activities of glutamine synthetase in roots of *S. breviflora*.

## Discussion

Grazing is one of the most important management practices in desert steppe, but its fragile ecological characteristics make soil and vegetation extremely sensitive to the disturbance of grazing livestock [8, 34]. Significant amounts of energy and material are removed by grazing disturbance, especially in heavily and overgrazed grassland [35, 36]. The absorption and utilization efficiency of plant nutrients can reflect the growth state and internal characteristics of plants. In low productivity ecosystems, grazing results in a decrease in soil nitrogen pools [37, 38]. In this study, the hypothesis that grazing intensity affects the activities of nitrogen assimilation related enzymes in plant roots and leaves was supported, and the results showed that changes in nitrogen assimilation related enzyme activities affect the nitrogen absorption and utilization efficiency of plants. This can be seen from the changes in total nitrogen content and nitrate nitrogen content in roots and leaves of *S. breviflora*. Different from our first hypothesis, not all the changes in enzyme activities related to nitrogen assimilation were consistent with the moderate interference hypothesis.

Nitrogen assimilation plays a crucial role in plant life activities, and directly affects the growth and development of plants [39]. Nitrate reductase (NR), glutamine synthetase (GS), glutamic acid synthetase (GOGAT), glutamic oxaloacetate transaminase (Got) and glutamic pyruvate transaminase (GPT) activities directly affect the nitrogen assimilation process of plants, and also affects the assimilation efficiency of nitrogen [40]. According to the correlation analysis results on total nitrogen content, nitrogen compound content and nitrogen assimilation related enzyme activities in the roots and leaves of *S. breviflora* (Table 1), our hypothesis that nitrogen content and nitrogen compound content in the leaves and roots of *S. breviflora* would be closely related to nitrogen assimilation related enzyme activities was generally supported, but not all the activities of nitrogen assimilation related enzymes were significantly positively correlated with the content of nitrogen compounds, which was different from the findings of previous studies [34, 41]. Because there are few studies on the activities of enzymes related to plant nitrogen assimilation in grassland, we have not even found relevant research reports, so here we further discuss and analyze the role of related enzymes in the process of plant nitrogen assimilation.

NR is a key enzyme in the process of nitrogen metabolism, and its activity is closely related to nitrogen assimilation capacity [42]. Part of the NO_3_^−^ absorbed by plant roots generates NH_4_^+^ under the catalysis of NR and NiR, and the other part is transferred to the leaves. Our results indicated that the heavy grazing treatment significantly reduced the transformation of NO_3_^−^ to NH_4_^+^ in the root system of *S. breviflora*, and the conversion of NO_3_^−^ to NH_4_^+^ in leaves was significantly reduced by grazing exclusion and heavy grazing, which affected the efficiency of nitrogen assimilation. A deficiency of this study is that it did not measure NH_4_^+^ and NiR in the roots and leaves of *S. breviflora*. Light and moderate grazing significantly increased NR activity in leaves, and it is inferred that the conversion efficiency of NO_3_^−^ to NH_4_^+^ is high, which may also be the reason for the high nitrogen content in *S. breviflora* leaves under light and moderate grazing.

Under the catalysis of GS and GOGAT, NH_4_^+^ forms glutamic acid and glutamine, and GS and GOGAT process are carried out simultaneously. Li et al. [34] studied the process of nitrogen assimilation in maize, and showed that the higher the activity of GS and GOGAT, the stronger the ability for nitrogen assimilation. Our research showed slightly different results in that there was a significant negative correlation between GS and total nitrogen content in roots and leaves of *S. breviflora*. That is, the higher the GS activity in roots and leaves, the lower the total nitrogen content in roots and leaves of *S. breviflora*. However, this finding is similar to the results of Fei et al. [43], who showed that GS activity did not follow the same trend as biomass and nutrient content. The reason may be related to the content of NH_4_^+^ in plant roots and leaves, because studies have shown that GS is more sensitive to low concentrations of NH_4_ ^+ 1^, and GS activity will be significantly reduced under high NH_4_^+^ [44]. In our study, there was a significant positive correlation between GOGAT activity and total nitrogen content in roots and leaves, which was the same as found in previous studies [41]. GOT and GPT are important transaminases in the process of nitrogen assimilation in plant roots and leaves. Wang et al. [34] showed that GOT and GPT activities were significantly positively correlated with total nitrogen content in leaves of plants, and our study found the same result, indicating that the higher GOT and GPT activities in roots and leaves of *S. breviflora*, the higher total nitrogen content. These results indicate that under light and moderate grazing disturbance, grassland plants can improve their nitrogen assimilation and utilization efficiency by regulating the increase of some nitrogen assimilation related enzyme activities (NR, GS, GOT and GPT).

The process of nitrogen assimilation in plants is complex, not only involving many enzymes, but also the amount of substrate produced affects the assimilation efficiency [44]. Our results showed that heavy grazing significantly reduced the content of soluble protein and amino acids in roots, and significantly increased the content of amino acids in leaves, and correlation analysis showed that this was related to the changes in GS, GOT, GPT and NR activities in roots and leaves. Therefore, we suggest that grazing exclusion and heavy grazing significantly affect the formation of substrate in the process of nitrogen assimilation. The content of proline could reflect the stress intensity of plants to a certain extent [45, 46]. Grazing can disturb the growth state and adaptability of plants, so it has been considered as a biological stress for most plant species [12]. Therefore, in our study, the higher proline content in roots and leaves of *S. breviflora* under grazing prohibition and heavy grazing showed that grazing prohibition and heavy grazing were not conducive to the normal growth and development of plants.

In addition to the change in enzyme activities related to nitrogen assimilation, the change in soil nitrogen content is also an important reason for the influence of grazing intensity on plant nitrogen absorption, as it particularly affects the content of ammonium nitrogen and nitrate nitrogen in rhizosphere soil. Our results differ from those of many studies which have shown that with increasing grazing intensity, the nitrogen content in soil increases gradually due to increased feces and urine deposition by livestock [47, 48]. We suggest that the reason for this difference is due to the different grazing methods. In this experiment, the livestock were fed in the experimental plot during the day, and were driven back to the shed at night to rest, and most of the feces and urine of livestock were left in the shed [21]. The experimental design of other studies has been that livestock feed and rest from morning to night in the experimental area, and livestock excreta are distributed in the experimental area, so the soil nitrogen content was relatively high. Based on the analysis of nitrogen assimilation related enzyme activities and nitrogen compound contents, we concluded that high nitrogen (no grazing) and low nitrogen (heavy grazing) environments were not conducive to nitrogen absorption and utilization in the roots and leaves of *S. breviflora*. Our study only systematically sampled, measured and analyzed *S. breviflora*, a constructive species in the desert grassland. We will further study the nitrogen assimilation mechanism of other plants in the desert grassland, so as to reveal the mechanism of plant nitrogen material cycle in grazing grassland.

## Conclusions

Our results showed that grazing disturbance can change the nitrogen use efficiency of grassland plants by regulating the activities of nitrogen assimilation related enzymes in leaves and roots. Light or moderate grazing disturbance is beneficial to the nitrogen assimilation of grassland plants, and can promote nitrogen cycling in the grassland ecosystem, which is consistent with the moderate disturbance hypothesis. These findings, combined with the reported moderate grazing disturbance, are conducive to the improvement of grassland vegetation diversity and productivity, indicating that the appropriate stocking rate plays an important role in the stability and sustainable utilization of the grassland ecosystem.

## Data Availability

The datasets used and/or analyzed during the current study are available from the corresponding author on reasonable request.

## Abbreviations

CK: No grazing
LG: Light grazing
MG: Moderate grazing
HG: Heavy grazing
NR: Nitrate reductase
GS: Glutamine synthetase
GOGAT: Glutamic acid synthetase
GOT: Glutamic oxaloacetic transaminase
GPT: Glutamic pyruvate transaminase
TN: Total nitrogen content
SP: Soluble protein
Pro: Proline
NN: Nitrate nitrogen
FAA: Free amino acid
Glu: Glutamate
Gln: Glutamine
Asp: Aspartic
Asn: Asparagines
